# Sustained antiviral insulin signaling during West Nile virus infection results in viral mutations

**DOI:** 10.3389/fcimb.2024.1492403

**Published:** 2024-11-27

**Authors:** Aditya B. Char, Chasity E. Trammell, Stephen Fawcett, Manish Chauhan, Yared Debebe, Nora Céspedes, Ryder A. Paslay, Laura R. H. Ahlers, Dharmeshkumar Patel, Shirley Luckhart, Alan G. Goodman

**Affiliations:** ^1^ School of Molecular Biosciences, College of Veterinary Medicine, Washington State University, Pullman, WA, United States; ^2^ Deptartment of Microbiology, Immunology, and Pathology, Colorado State University, Fort Collins, CO, United States; ^3^ Department of Entomology, Plant Pathology, and Nematology, College of Agricultural and Life Sciences, University of Idaho, Moscow, ID, United States; ^4^ Center for ViroScience and Cure, Laboratory of Biochemical Pharmacology, Department of Pediatrics, Emory University School of Medicine and Children’s Healthcare of Atlanta, Atlanta, GA, United States; ^5^ Department of Biological Sciences, College of Science, University of Idaho, Moscow, ID, United States; ^6^ Paul G. Allen School of Global Health, College of Veterinary Medicine, Washington State University, Pullman, WA, United States

**Keywords:** fruit fly, *Drosophila melanogaster*, mosquito, vector, IIS, evolution, flavivirus, *Culex quinquefasciatus*

## Abstract

Arthropod-borne viruses or arboviruses, including West Nile virus (WNV), dengue virus (DENV), and Zika virus (ZIKV) pose significant threats to public health. It is imperative to develop novel methods to control these mosquito-borne viral infections. We previously showed that insulin/insulin-like growth factor-1 signaling (IIS)-dependent activation of ERK and JAK-STAT signaling has significant antiviral activity in insects and human cells. Continuous immune pressure can lead to adaptive mutations of viruses during infection. We aim to elucidate how IIS-signaling in mosquitoes selects for West Nile virus escape variants, to help formulate future transmission blocking strategies. We hypothesize that passage of WNV under activation of IIS will induce adaptive mutations or escape variants in the infecting virus. To test our hypothesis, WNV was serially passaged through *Culex quinquefasciatus* Hsu cells in the presence or absence of bovine insulin to activate IIS antiviral pressure. We sequenced WNV genes encoding for E, NS2B, NS3, and NS5 and identified variants in *E* and *NS5* arising from IIS antiviral pressure. In parallel to the genetic analyses, we also report differences in the levels of virus replication and Akt activation in human cells and mosquitoes using virus passaged in the presence or absence of insulin. Finally, using adult *Culex quinquefasciatus*, we demonstrated the enhancement of immune response gene expression in virus-infected mosquitoes fed on insulin, compared to control. Notably, virus collected from insulin-fed mosquitoes contained a non-synonymous mutation in *NS3*. These results contribute towards achieving our long-term goal of manipulating mosquito IIS-dependent antiviral immunity to reduce WNV or other flavivirus transmission to mammalian hosts.

## Introduction

West Nile Virus (WNV) is a flavivirus belonging to the Japanese encephalitis serogroup. The first case of WNV in North America was recorded in New York in 1999 (WNV-NY99). Within 10 years the virus had spread across the continent (Kramer et al., 2019). This pathogen is transmitted through birds and the bite of infected mosquitoes, and climate change is expected to exacerbate its threat. As global temperatures rise, the latitudes at which the virus can be harbored in mosquitoes, and the ranges these mosquitoes can inhabit, will expand (Alaniz et al., 2018). Currently, only a small percentage of infected people develop the deadly neuroinvasive form of this disease. The emerging threat of WNV is that millions of people will be at risk of infection in the near future. It is necessary, therefore, to increase our understanding how this pathogen interacts with its vectors and hosts to combat this threat. Using the *Drosophila melanogaster* model, a novel antiviral immune pathway in mosquitoes was elucidated that is stimulated by ingested mammalian insulin (Ahlers et al., 2019). This pathway is also relevant in human cells where it interacts with endothelin signaling to recruit antiviral immunity (Trammell et al., 2023). The study presented here is aimed towards understanding how this antiviral mechanism in the mosquito vector affects the genome and virulence of WNV.

*D. melanogaster* has been previously used to show that insulin-mediated induction of the Janus kinase/signal transducer and activator of transcription (JAK/STAT) signaling plays a vital role in host survival and immunity to WNV. We reported that insulin-mediated JAK/STAT signaling is conserved in *Culex quinquefasciatus*, a primary vector of WNV (Ahlers et al., 2019). JAK/STAT signaling is mediated by the domeless receptor cascade in insects (Agaisse and Perrimon, 2004). Briefly, the binding of endogenous insulin or insulin-like peptides (ilps) to insulin-like receptor (InR) triggers the phosphorylation of Akt and Extracellular Signal-Regulated Kinase (ERK). Phosphorylated ERK upregulates the expression of the protein upd2/3, which in turn activates the domeless receptor. The domeless receptor signals through hopscotch and leads to activation of the transcription factor Stat92E that induces antiviral effector genes, such as *vir-1* and *Vago2* (Luckhart and Riehle, 2007; Ahlers et al., 2019). In parallel, the phosphorylation of Akt induces the phosphorylation of the forkhead transcription factor FoxO. This prevents FoxO from being localized to the nucleus. The nuclear localization of FoxO induces the expression of *Dicer 2* (*Dcr2*), *Argonaute 1* (*AGO1*), and *AGO2* components of the RNA interference (RNAi) pathway (Wang et al., 2014; Spellberg and Marr, 2015). Thus, stimulation of InR activates JAK/STAT signaling while suppressing RNAi.

Mosquito InR recognizes a family of peptides called insulin-like peptides (ilps), as well as mammalian insulin (Wu and Brown, 2006; Yamaguchi et al., 1995), biology that is enabled by the evolutionary conservation of these insect and mammalian peptides (Sajid et al., 2011). The ingestion of mammalian insulin has been found to reduce the titer of WNV in infected fruit flies and mosquitoes (Ahlers et al., 2019). While birds are a natural WNV reservoir and infectious host, they have low levels of serum insulin that do not correlate with glucose levels (Holmes et al., 2001). In contrast, mammals have high insulin levels and exhibit insulin-dependent glucose uptake (Polakof et al., 2011). *Cx. quinquefasciatus* mosquitoes are opportunistic feeders with, in some cases, 52.5% of their bloodmeals derived from mammals (Molaei et al., 2007), a fact that underlies the relevance of the *in vitro* and *in vivo* responses of this species to mammalian insulin (Ahlers et al., 2019).

Across multiple species, mosquitoes lack an adaptive immune response and primarily control viral infection by the RNAi pathway (Olson and Blair, 2015; Ahlers et al., 2019). The ingestion of insulin by mosquitoes stimulates antiviral genes through JAK/STAT signaling while suppressing the RNAi response (Sim and Denlinger, 2008). Understanding how JAK/STAT affects virus mutation could be important in developing methods to control this pathogen. This type of mitigation strategy has been suggested for developing combinational antiviral therapies for HIV; mathematical models simulated the relationship between HIV intra-host genotypes and antiviral drugs. These models would predict the optimal time to change the antiviral drug to avoid the development of resistance and virus escape (Hernandez-Vargas et al., 2011). A similar approach might be used to predict patterns of mutation in flaviviruses and how these viral mutations persist in a population of mutant virus genomes during antiviral treatment.

The effects of antiviral immune pathways on the evolution of flaviviruses is well documented, as viruses can develop mechanisms to hinder these immune responses. For example, the NS5 protein of the NY99 strain of WNV (WNV-NY99) disrupts JAK/STAT signaling. This is in contrast to the WNV-Kunjin (Kun) NS5 protein, in which a single peptide change results in weak JAK/STAT antagonism compared to that of WNV-NY99 NS5 (Laurent-Rolle et al., 2010). Additionally, WNV-NY99 delays IRF-3 signaling which is an important transcription factor for innate immune responses (Fredericksen and Gale, 2006). The diverse range of hosts that the *Flaviviridae* family infects places unique pressure on the evolution of these viruses. The genomes of viruses that infect only vertebrate or invertebrate hosts can be distinguished by their unique codon usage and dinucleotide patterns (Lobo et al., 2009). In mosquitoes, the RNAi immune response drives WNV diversification as rare genotypes that can escape sequence-specific short interfering RNAs (Pesko and Ebel, 2012). Given that the ingestion of insulin suppresses this pathway, WNV will be subjected to a novel selective pressure from IIS-mediated immunity.

This study aims to catalogue the impact of the insulin-mediated antiviral immune response on the genome of the NY99 strain of WNV. The immune response stimulated by the insulin receptor in insects such as *D. melanogaster* improves survival during infection with the WNV-Kun (Ahlers et al., 2019). In the work presented here, we demonstrate that IIS-mediated selection is equally significant against the pathogenic WNV-NY99 strain. Key components of IIS, including the insulin receptor and ilp7, improve survival during infection in *D. melanogaster*. Additionally, insulin stimulation reduces WNV-NY99 load in insect cells *in vitro* as well as in adult fruit flies. To investigate the impact of continuous IIS-mediated selection pressure on WNV, we serially passaged WNV-NY99 through *Culex quinquefasciatus* Hsu cells and performed sequenced WNV genes after each passage to catalog genetic changes. Under IIS selection pressure, we identified unique viral genotypes whereby WNV appeared to lose its capacity to manipulate Akt signaling in mammalian cells. *In vivo* infection in adult *Cx. quinquefasciatus* under IIS pressure resulted in a mutation in the NS3 virus protein, with molecular dynamics simulations predicting impaired NS3 protein function. Our findings reveal that WNV under IIS-mediated selection pressure develops distinctive genotypic and phenotypic characteristics, highlighting the importance of this antiviral immune pathway during WNV infection in the mosquito vector.

## Results

### Insulin reduces WNV-NY99 infection in *D. melanogaster* and insect cell lines

**Figure 1:**
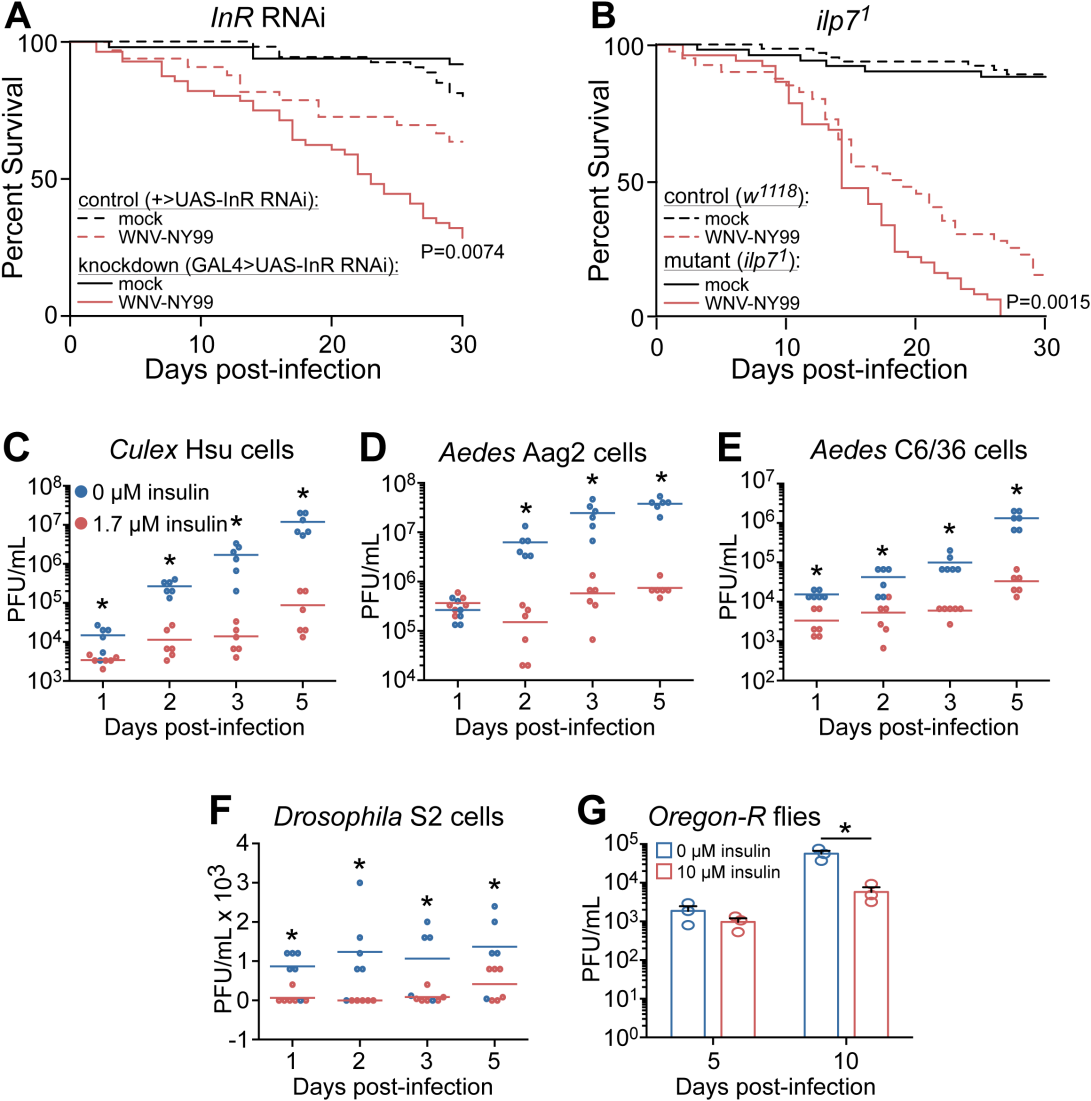
Insulin signaling protects against mortality to WNV-NY99 infection and reduces virus replication. **(A)** *InR* knockdown (mock, n = 94; WNV, n = 110) and **(B)** *ilp7^1^* mutant (mock, n = 100; WNV, n = 102) *D*. *melanogaster* are more susceptible to WNV-NY99 (2,000 PFU/fly) compared to genetic controls (+>UAS-InR RNAi: mock, n = 104; WNV, n = 99; *w^1118^*: mock, n = 63; WNV, n = 80). Significance compared to infected control *D. melanogaster* is indicated (Log-rank test). **(C–F)** WNV-NY99 (MOI=0.01 PFU/cell) titer is reduced in **(C)** *Cx. quinquefasciatus* Hsu, **(D)** *Ae. aegypti* Aag2, **(E)** *Ae. albopictus* C6/36, and **(F)** *D*. *melanogaster* S2, cells primed with 1.7 μM bovine insulin for 24 h prior to infection. Note the use of a linear scale. **(G)** WNV-NY99 (dose 2,000 PFU/fly) replication is reduced in adult *D*. *melanogaster* fed 10 μM bovine insulin (Unpaired t-test; *p < 0.05).

In insects, InR, ilp, and their downstream signaling pathways have multiple evolutionarily conserved functions including larval development, regulation of feeding and immune response regulation (Grönke et al., 2010; Darby and Lazzaro, 2023). To demonstrate the importance of IIS in *D. melanogaster* during WNV-NY99 infection we knocked-down *InR* by RNAi and used mutant flies deficient in *ilp7*. Previous studies have found that these genes were similarly important for survival against the attenuated WNV-Kun strain (Ahlers et al., 2019). Unlike other fruit fly ilps, ilp7 is conserved in mosquitoes (Grönke et al., 2010). We infected control and *InR* knockdown or *ilp^7^* mutant *D. melanogaster* with 2,000 PFU of WNV-NY99 by intrathoracic injection and monitored survival for 30 days. These results show that the knockdown of InR significantly increased the susceptibility of flies to WNV-NY99 infection (**Figure 1A**). Similarly, mutant flies lacking *ilp^7^* were more likely to succumb to WNV-NY99 infection (**Figure 1B**).

To show the importance of insulin signaling during WNV-NY99 infection in insect cells, we treated multiple mosquito cell lines and *D. melanogaster* S2 cells with 1.7 μM bovine insulin prior to WNV infection (Esmann, 1963; Kang et al., 2008; Surachetpong et al., 2009; Xu et al., 2013; Ahlers et al., 2019). Similar to previous findings using WNV-Kun (Ahlers et al., 2019), we found that insulin treatment reduced WNV-NY99 viral load throughout infection (**Figures 1C–G**). We showed that the antiviral activity of IIS-mediated immunity is functional in mosquito cell lines from *Culex* (**Figure 1C**) and *Aedes* species (**Figures 1D, E**), as well as *Drosophila* S2 cells (**Figure 1F**). We next tested if ingested insulin reduced WNV-NY99 infection in adult *D. melanogaster*. Fruit fly food was supplemented with 10 μM bovine insulin or vehicle control. By 10 dpi, fruit flies in the insulin-fed group had lower levels of WNV compared to control flies (**Figure 1G**). Taken together these data show that insulin signaling reduces levels of the pathogenic strain of WNV in adult fruit flies and insect cells, supporting our previous results using WNV-Kun (Ahlers et al., 2019).

### Mutations arise during serial passage of WNV-NY99 in insulin-treated *Cx. quinquefasciatus* Hsu cells

**Figure 2:**
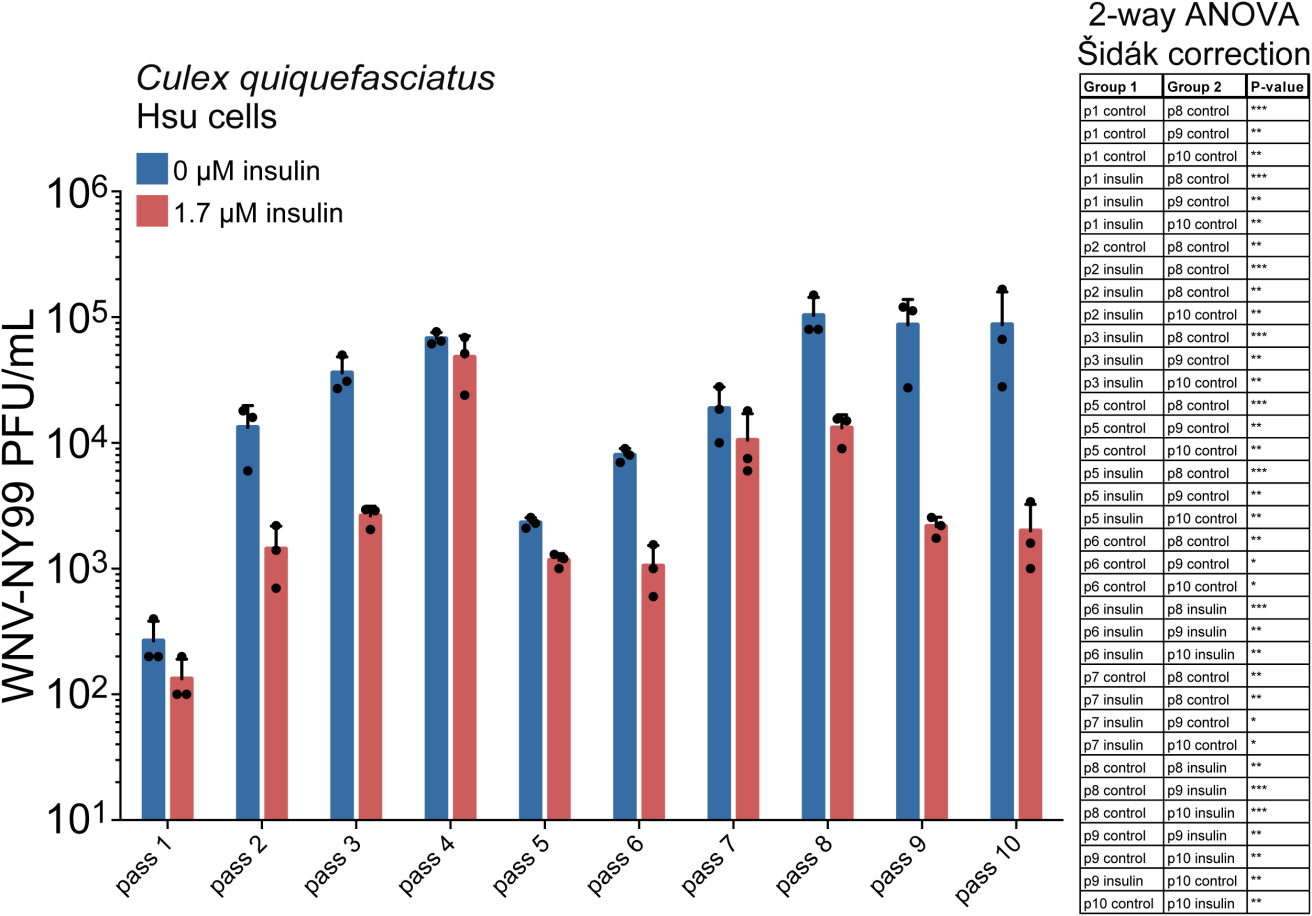
Replication of WNV-NY99 serially passaged in Hsu cells. Hsu cells were treated with 1.7 μM insulin or vehicle control, then infected with WNV-NY99 (MOI=0.001 PFU/cell). Media from infected cells was collected 5 dpi. Titer was determined by plaque assays. Data are the average titer of three biological replicates (2-way ANOVA with Šidák correction; *p < 0.05, **p < 0.01, ***p < 0.001).

The effects of immunity on inhibiting virus growth can induce the development of viral evasion strategies (Tenthorey et al., 2022). For instance, the receptor binding domain (RBD) of SARS-CoV-2 was reported to develop escape mutations when the virus was cultured with anti-RBD antibodies (Greaney et al., 2021). Similarly, the envelope protein of HIV is known to develop mutations in conserved sites targeted by therapeutic broadly neutralizing antibodies (Dingens et al., 2017). Individual immune pathways exert unique selective pressures in comparison to these pathways working in concert (Mongelli et al., 2022). We hypothesized that serial passage of WNV during insulin stimulation would select for virus sequence variants, since insulin stimulates JAK/STAT immunity while suppressing the RNAi response (Ahlers et al., 2019). WNV infection alone was not sufficient to stimulate a JAK/STAT response (Ahlers et al., 2019). We performed serial passage of WNV-NY99 in Hsu cells under sustained insulin-mediated selection, with cells infected with virus from the previous passage for 10 passages. Cells were primed with media containing 1.7 μM bovine insulin or vehicle control 24 hours prior to infection with virus. Virus load was analyzed at each passage. We observed that insulin treatment significantly reduced the virus load in Hsu cells after 8-10 passages (**Figure 2**). Viral titers under insulin treatment at early passages 1-3 and 5-7 were also signifcantly lower than titers in control cells at passages 8-10. These results suggested that the effects of insulin were greater as passage number increased, suggesting that treatment may be affecting the virus.

To test if sustained insulin-mediated selection was associated with changes in the WNV-NY99 genome, we performed Sanger sequencing of WNV genes *E, NS2B, NS3*, and *NS5* in virus collected from infected cells from passages 5 and 9 (**Table 1**). Previous studies have shown that these genes are prone to mutation in other circumstances of selection (Jerzak et al., 2007; Ebel et al., 2011). The proteins coded by these viral genes play important functions during the establishment of the disease, specifically virus entry, genome replication, and immunity, and they interact with the PI3K/Akt/ERK signaling axis (Scherbik and Brinton, 2010; Albentosa-González et al., 2021; Xie et al., 2021) that is downstream of insulin receptor signaling. The E protein is an important structural protein that is a major target of the host immune response (Arjona et al., 2007). The proper conformations of the E protein in many WNV variants, including NY99, require an N-linked glycosylation at residues 154-156. A study examining this glycosylation site reported that mice infected with WNV-NY99 mutants lacking this motif were less likely to develop the neuroinvasive disease (Shirato et al., 2004). The NS3 protein is a multi-functional molecule and plays many important roles in WNV infections. It has helicase activity, nucleotide triphosphatase activity and in conjunction with NS2B, it acts as a serine protease (Aleshin et al., 2007). This protease cleaves the other non-structural proteins from the viral polyprotein during infection. The NS2B/NS3 protease of dengue virus, another flavivirus, is known to disrupt IFN type I production (Setoh et al., 2017). The NS5 protein of WNV-NY99 is a potent antagonist of type I interferon activity. Research comparing the NS5 proteins of WNV-NY99 and WNV-Kun showed that the NY99 protein was 30-fold more effective at suppressing IFN-mediated JAK/STAT signaling (Laurent-Rolle et al., 2010). With Sanger sequencing, we examined sequences only for *E, NS2B, NS3*, and *NS5*. Future studies with RNA sequencing of the WNV genome will reveal mutations associated with other viral gene products.

**Table 1:** Codon usage analysis of WNV-NY99 *envelope* (*Env*) and *NS5* genes.

Treatment	Passage	Gene	SNP Position on consensus	Codon Change (WT/SNP)	Silent mutation?	Amino acid	Codon usage fraction in *Culex quinquefasciatus* (SNP/WT)*	Codon usage fraction in *H. sapiens* (SNP/WT)*
1.7 μM insulin	Pass 5	*Env*	C1603A	cuC/cuA	Yes	Leucine	0.61	0.37
1.7 μM insulin	Pass 5	*Env*	C549U	guC/guU	Yes	Valine	1.22	0.76
1.7 μM insulin	Pass 5	*NS5*	G1861A	cuG/cuA	Yes	Leucine	0.16	0.18
1.7 μM insulin	Pass 9	*Env*	C549U	guC/guU	Yes	Valine	1.22	0.76

*Frequency of amino acid per thousand.

In passage 5 and 9 virus, we did not detect any mutations in the *NS2B* and *NS3* genes compared to the original stock genome. However, we did detect mutations in the *envelope* and *NS5* genes in replicates of insulin-treated Hsu cells, with a single synonymous C to T transition at position 549 of the *envelope* gene that persisted from passage 5 to 9. Mutations were not observed in replicates of control cells nor did any mutation persist in these cells from passage 5 to 9. We calculated the codon usage as a fraction of frequency of amino acid (SNP) per thousand over frequency of amino acid (wild-type) per thousand in *Cx. quinquefasciatus* and *H. sapiens*. Synonymous codons are not used equally within organisms and the abundance tRNAs for their specific codons can affect translation rate, causing ribosome pausing while the less abundant tRNAs are recruited. This could also affect the timing and pattern of protein folding (Jiang et al., 2023). Codon usage fractions less than one represent mutant codons that are less abundant in the organism while values greater than one represent mutant codons that are more abundant (Jiang et al., 2023). Codon usage fractions were calculated using values from the Kasuza codon usage database (www.kazusa.or.jp/codon) (Nakamura et al., 2000). For example, we found that the codon frequency for *envelope* C1603A and *NS5* G1861A mutations was ~2-5-times lower than wild-type (**Table 1**, columns 8-9), suggesting decreased tRNA codon availability for the transcript variant that could affect translation of the WNV polypeptide in both *H. sapiens* and *Cx. quinquefasciatus*. Additionally, we found that the codon frequency for the persistent *envelope* C549U mutation was 22% higher than wild-type in *Cx. quinquefasciatus*, suggesting that there is increased tRNA availability for this codon variant. Increased tRNA availability could also lead to increased translation of the WNV polypeptide and counteract insulin-mediated antiviral effects. Together, these results suggest that serial passage of WNV-NY99 in *Cx. quinquefasciatus* cells under insulin pressure induces mutations in the WNV genome that could affect virus replication under treatment. RNA sequencing in future studies could be used to determine proportional abundance of mutants found within a viral population.

**Figure 3:**
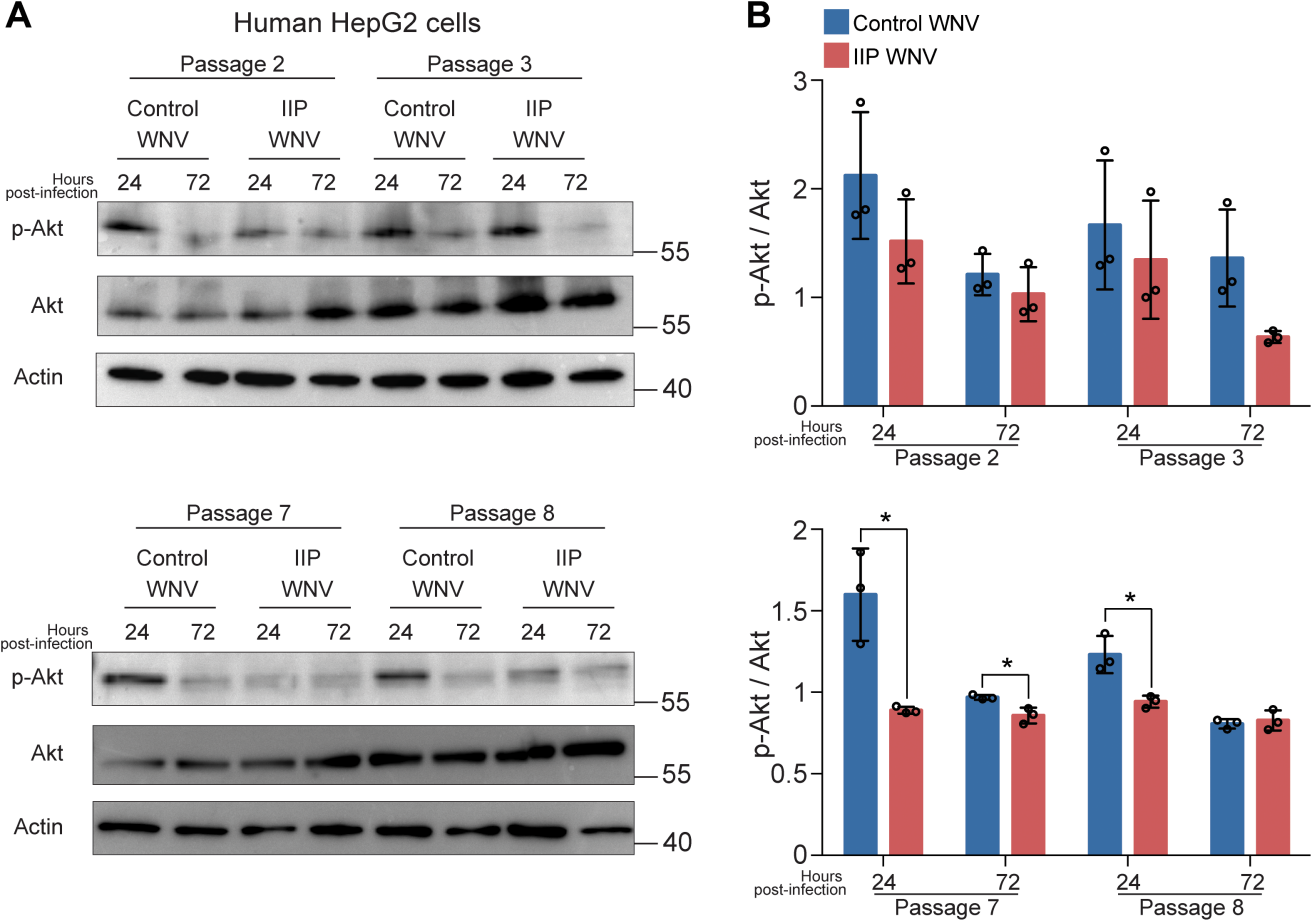
Serial passage under IIS-mediated immunity reduces Akt signaling in HepG2 cells. **(A)** HepG2 cells were infected with WNV-NY99 from Hsu passage treatment groups. The supernatant from the wells of each treatment group are pooled before infecting the HepG2 cells. Cells are lysed at 24 and 72 hpi. Control WNV is collected from Hsu cells in 0 μM insulin media. IIP WNV is collected from Hsu cells in 1.7 μM media. **(B)** Quantification of pAKT intensity compared to total AKT intensity from three independent immunoblots in **(A)** (Unpaired t-test; *p < 0.05).

**Figure 4:**
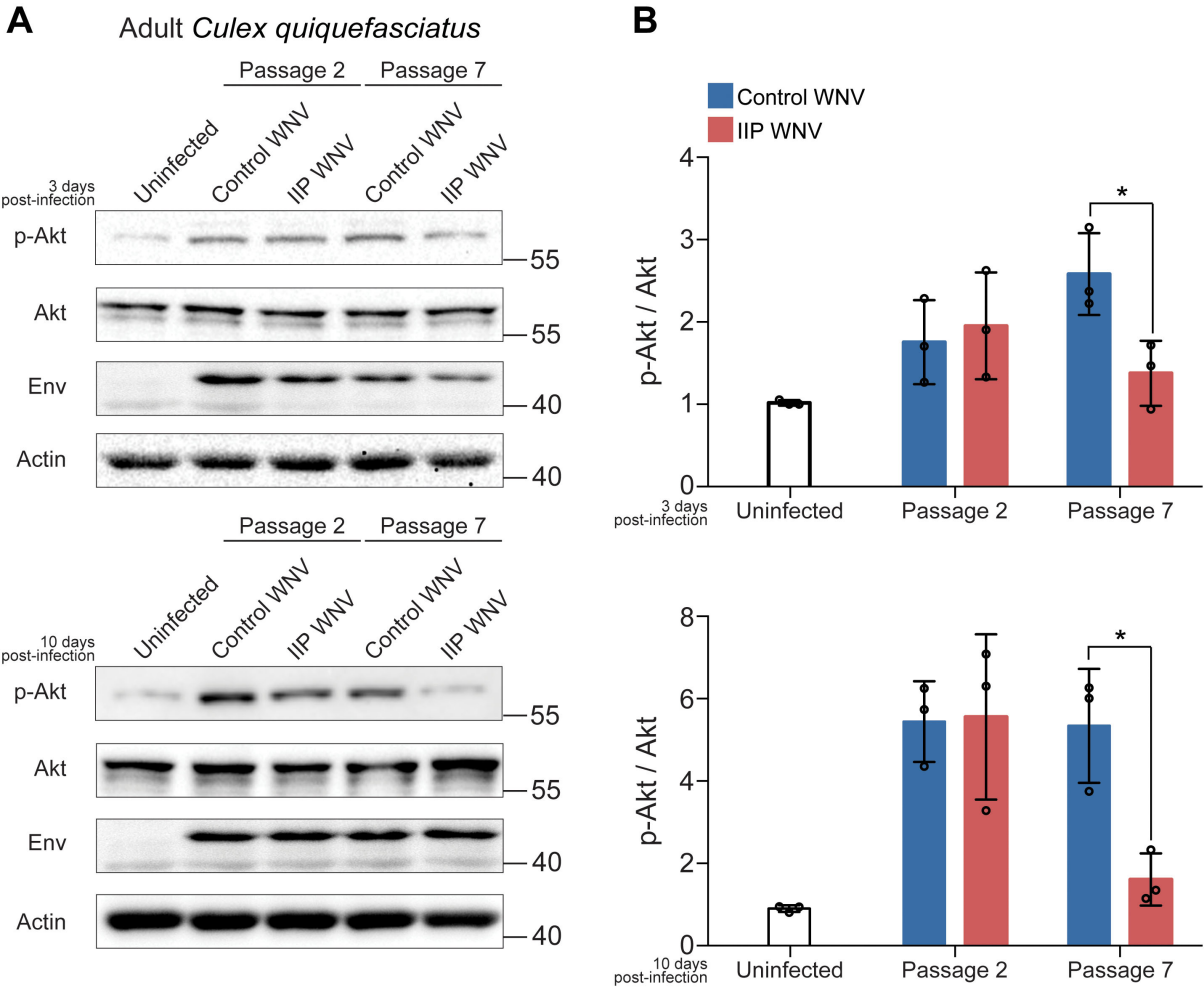
Serial passage under IIS-mediated immunity reduces Akt signaling in adult *Cx. quinquefasciatus*. **(A)** *Cx. quinquefasciatus* were bloodfed WNV-NY99 from control or insulin-treated Hsu cells at virus passage 2 or 7. At 3 or 10 dpi, five groups of five mosquitoes were homogenized in RIPA buffer and equal amounts of protein from each biological replicate were pooled for immunoblot analysis. **(B)** Quantification of pAKT intensity compared to total AKT intensity from three independent immunoblots in **(A)** (Unpaired t-test; *p < 0.05).

We next examined human cellular and adult mosquito responses to control WNV or insulin-selected WNV. Given that flaviviruses stimulate Akt phosphorylation to promote cell survival and abrogate pro-apoptotic signaling (Dunn and Connor, 2012), we infected human liver HepG2 cells and adult *Cx. quinquefasciatus* with passaged WNV and used western blotting to quantify Akt activation (phosphorylation) in infected cells (**Figure 3**) and mosquitoes (**Figure 4**). This cell model is relevant based on our earlier work with HepG2 cells showing that endothelin signaling and associated Akt activation are induced by insulin stimulation to control WNV replication (Trammell et al., 2023). Furthermore, insulin receptor signaling in mosquitoes also activates Akt (Ahlers et al., 2019; Trammell et al., 2022). We compared Akt phosphorylation between HepG2 cells infected with control or insulin immune pressured (IIP) WNV. In passages 2 and 3 there was no difference in Akt phosphorylation between the treatment groups at 24 and 72 hpi. However, at passages 7 and 8, while HepG2 cells infected with control WNV displayed Akt phosphorylation at 24 hpi, cells infected with IIP WNV displayed lower Akt phosphorylation at 24 hpi (**Figure 3**). In adult *Cx. quinquefasciatus*, there was reduced Akt phosphorylation at 3 and 10 dpi following infection with IIP WNV from passage 7 compared to control WNV. However, there was no difference in Akt phosphorylation following mosquito infection using control or IIP WNV from passage 2 (**Figure 4**). Together, these results suggest that the insulin-induced WNV mutations due to serial passage affect Akt phosphorylation in human HepG2 cells and adult *Cx. quinquefasciatus*. It would be valuable to test at which passage WNV loses its ability to activate Akt in cells and mosquitoes. Future studies could also examine how insulin in *Cx. quinquefasciatus* bloodmeals affects replication of control WNV-NY99 and IIP WNV-NY99, as well as how these viruses affect mortality in *D. melanogaster*.

### Insulin activates mosquito immunity during WNV infection

**Figure 5:**
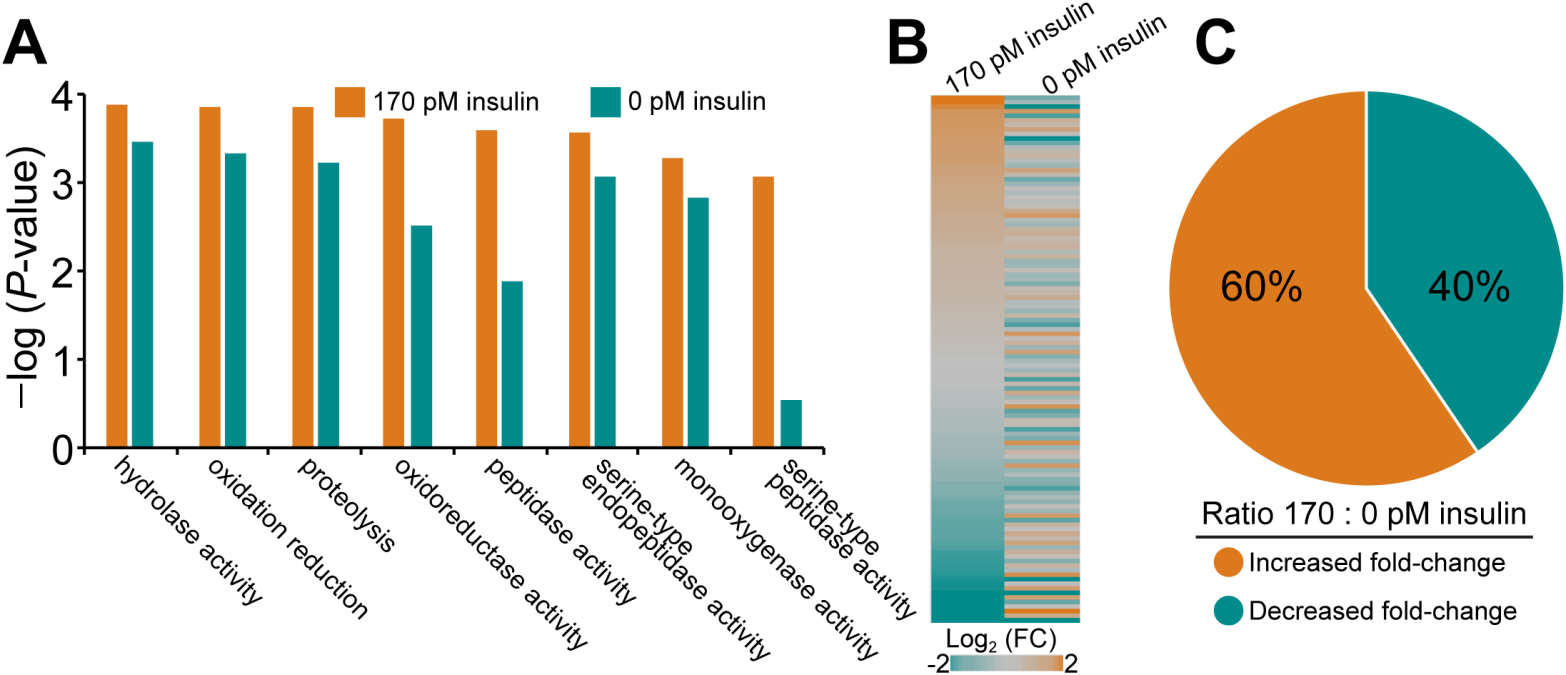
Ingested insulin induces defense responses in *Cx. quinquefasciatus*. **(A)** Gene set enrichment analysis (GSEA) using RNA-seq datasets from WNV-Kun-infected mock- and insulin-treated mosquitoes at 10 dpi shows increased enrichment of defense response GO categories (Reid et al., 2018). **(B)** Heatmap using geneset described previously as up-regulated during WNV-NY99 infection (Paradkar et al., 2015). **(C)** Ratio of gene expression in **(B)** showing insulin up-regulates a majority of WNV-induced genes.

Our data from Hsu cells showed that serial passage of WNV-NY99 under insulin-mediated selection was associated with mutations in the WNV *envelope* and *NS5* genes and altered host responses. Accordingly, we sought to examine host responses to infection under insulin selection in *Cx. quinquefasciatus*, the primary vector of WNV in North America (Rochlin et al., 2019). We infected female mosquitoes with WNV-Kun in the presence or absence of 170 pM bovine insulin in the bloodmeal (Ahlers et al., 2019). At 10 dpi, total RNA was collected from groups of five mosquitoes in each treatment group for RNA sequencing. Using these data, we performed Gene Set Enrichment Analysis (GSEA) to analyze gene expression patterns. Gene ontology (GO) categories related to host defense and immunity (Reid et al., 2018) were more enriched in WNV-Kun infected mosquitoes provisioned with 170 pM insulin relative to controls. For example, insulin fed mosquitoes showed significant enrichment of genes in the serine-type peptidase GO category (**Figure 5A**). Genes within this category have been linked to antiviral responses in mosquitoes against dengue virus (DENV). Multiple flaviviruses show antagonistic activity against the expression of a specific serine protease (Colpitts et al., 2011). Further, the oxidoreductase category includes genes that reduce Zika virus load in mosquitoes. This GO category is highly enriched in insulin-fed mosquitoes during WNV-Kun infection (Bottino-Rojas et al., 2018) as well as a similar GO category, response to oxidative stress, in *D. melanogaster* S2 cells treated with insulin during WNV-Kun infection (Trammell et al., 2023). We also analyzed the RNAseq data using a gene set derived from *Cx. quinquefasciatus* infected with WNV-NY99 (Paradkar et al., 2015) to generate a heatmap representing the fold change in gene expression compared to uninfected controls. We observed that a majority (60%) of genes were upregulated in infected mosquitoes that were blood fed 170 pM of insulin compared to vehicle control (**Figures 5B, C**; **Supplementary Table 1**).

**Figure 6:**
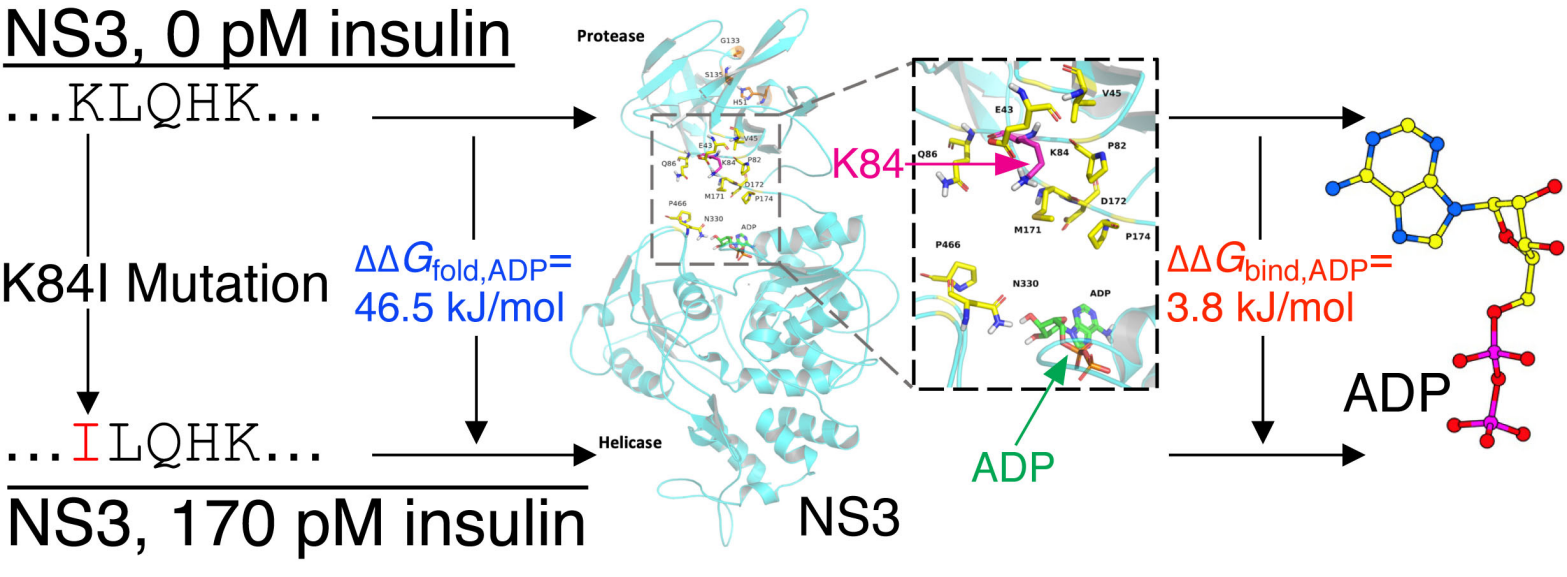
Molecular modeling of the NS3 K84I mutation. Modeling of a non-synonymous mutation in WNV-Kun following one passage (10 dpi) in *Cx. quinquefasciatus* fed 170 pM insulin indicates that the mutant NS3 K84I protein is less stable in the presence of ADP (ΔΔG_fold,ADP_ > 0) and may reduce ADP binding (ΔΔG_bind,ADP_ > 0).

We next used these RNAseq data from WNV-Kun-infected *Cx. quinquefasciatus* that were fed insulin or buffer control to map the data to the consensus sequence for WNV-Kun (GenBank Accession KX394396.1). We identified a non-synonymous mutation in the *NS3* gene (K84I) of WNV-Kun following a single virus passage in insulin-treated mosquitoes (**Figure 6**). The mutation was in the N-terminal protease domain close to ADP binding sites in the C-terminal helicase domain. Molecular dynamics simulations predicted that the K84I mutation affects ADP binding free energy (ΔΔG_bind,ADP_) and the folding free energy of the NS3-ADP complex (ΔΔG_fold,ADP_). We observed a small change in binding affinity (ΔΔG_bind,ADP_ = +3.8 kJ/mol) and observed a significant change in stability of the NS3-ADP complex (ΔΔG_fold,ADP_ = +46.5 kJ/mol). The K84 residue forms an H-bond with residue E43. A prior study examining the effects of amino acid changes on the stability of protein complexes suggested that ΔΔG values of ±8.4 kJ/mol and beyond represent a significant change. This value was determined by comparing free energy change predictions by simulations against experimental values (Patel et al., 2021). The K84I mutation lost this H-bond interaction which might affect the stability of the structure. This mutation was not detected in virus from infected control mosquitoes.

In summary, we demonstrated that IIS-mediated selection is associated with mutations in the WNV-NY99 genome both *in vitro* and *in vivo*. Additionally, insulin enhances the expression of immune genes that are predicted to contribute to host defenses against WNV. These promising findings encourage further investigation of the insulin-dependent antiviral pathways in the mosquito vector and how these shape the evolution of the WNV genome.

## Discussion

Previous studies showed that insulin signaling activates important antiviral immune pathways in both *D. melanogaster* and mosquitoes (Trammell et al., 2023; Xu et al., 2013; Ahlers et al., 2019). In the study presented here, we corroborated our previous findings in *D. melanogaster* using the attenuated Kunjin strain of WNV with the pathogenic WNV-NY99 strain. Notably, knockdown or ablation of components of the IIS-mediated immune pathway was detrimental to *D. melanogaster* survival during WNV-NY99 infection. Additionally, insulin priming prior to infection was found to reduce virus load in insect cell lines as well as in adult fruit flies.

The synonymous WNV mutations recorded in this study were seen in the genes coding for the envelope and NS5 proteins. The engineering of synonymous mutations in the same genes of other flaviviruses like Zika virus (ZIKV) and DENV has been associated with attenuation. For example, codon optimization was used to alter the sequence of the *envelope* gene of DENV and the *NS3* and *NS5* genes of ZIKV. These silent mutants have reduced fitness and virulence in a mosquito cell line and mice (Chin et al., 2023). We postulate that the occurrence of silent changes in the genes of WNV over sustained insulin-mediated selection could lead to changes in virulence. We have reported multiple synonymous mutations confined to WNV under insulin-mediated selection pressure. This included a synonymous mutation in the *envelope* gene that persisted in passages 5 and 9 and a synonymous mutation in sequence of the *NS5* gene encoding the C-terminal region that functions in RNA-dependent RNA polymerase activity required for viral replication (Brinton, 2014).

Our data from liver HepG2 cells and adult *Cx. quinquefasciatus* infected with serially passaged WNV revealed a divergence in the host response using WNV from early and late passages. Specifically, insulin-selected WNV from later passages did not elicit Akt phosphorylation to levels observed in cells or mosquitoes infected with control WNV from the same passages. Since late passage control WNV showed similar Akt phosphorylation levels to early passage WNV, we do not attribute this change to the serial passaging procedure itself. Accordingly, we propose that WNV manipulation of Akt signaling was lost due to sustained insulin-mediated selection on the virus genome. This would be detrimental to the virus as apoptosis of host cells must be prevented at early stages to avoid interruption of the virus replication cycle (Barber, 2001). Furthermore, while reduced Akt phosphorylation may lead to reduced JAK/STAT-mediated antiviral immunity, this could be compensated through an increase in another form of antiviral immunity, such as RNAi (Trammell et al., 2022). Conversely, the loss in Akt phosphorylation could be caused by the physiological effects of insulin priming on Hsu cells. The application of insulin itself stimulates the phosphorylation of Akt (Ahlers et al., 2019). Over the course of passaging, high levels of Akt phosphorylation induced by insulin could have supported, and even selected for, the growth of WNV variants that were unable to activate the PI3K/Akt pathway.

In this study we showed that insulin-mediated selection pressure*, in vitro and in vivo*, affects the genome of WNV-NY99. Additionally, we showed that IIS activation in the presence of WNV-NY99 infection enhances known defense responses of the mosquito vector. These data suggest that targeting IIS in the mosquito is a viable route for reducing WNV incidence and transmission in the field. There is a need for alternative approaches to WNV mitigation due to the drawbacks of currently employed strategies. For example, strategies that use *Wolbachia* to reduce viral infection in mosquitoes (Dodson et al., 2017; Thomas et al., 2018; Koh et al., 2019) have been shown to enhance WNV infection (Dodson et al., 2014).

To support our *in vitro* studies with Hsu cells, we examined the effects of insulin provisioning on WNV in *Cx. quinquefasciatus*. A single passage *in vivo* in insulin-fed mosquitoes was associated with the appearance of a non-synonymous mutation in the WNV *NS3* gene. Our molecular dynamics simulation predicted the thermodynamic stability of the mutant NS3 K84I protein in terms of the free energy difference (ΔΔG) in the presence of ADP, compared to the wild type. The positive value of ΔΔG indicates that the mutant K84I protein has impaired ADP binding affinity while the mutant protein-ADP complex is less stable (Li et al., 2020). ADP plays an important role in the function of the NS3 helicase given that the translocation of the protein on RNA is ATP-dependent. The replacement of ATP with ADP, as a result of hydrolysis, would lead to the favorability of particular protein confirmation states (Despins et al., 2010; Pérez-Villa et al., 2015). A reduction in the stability of the NS3-ADP complex, therefore, could result in the mutant K84I protein being functionally sub-optimal.

Our understanding of other flaviviruses and other pathogens could benefit from the findings of this study. Despite the reduction in virus titer seen in insulin-fed mosquitoes we do not recommend the feeding of insulin itself to mosquitoes given that this has been shown to enhance mosquito infection with malaria parasites (Pakpour et al., 2012). A more effective strategy could leverage the link between the insulin receptor and immune pathways to simultaneously activate RNAi and JAK/STAT signaling. The immune response produced by this combination was found to reduce the load of ZIKV in live mosquitoes to a greater degree than either pathway alone (Trammell et al., 2022).

Multiple components of the immune system are compromised in diabetic patients, leading to worse prognoses during viral infections (Daryabor et al., 2020). A recent meta-analysis of studies on WNV and DENV reported that diabetes was a significant risk factor for developing severe disease (Lu et al., 2024). Hyperglycemia has been suggested as the mechanism behind the increased risk; however, the precise cause remains unclear (Berbudi et al., 2020; Weng et al., 2021). The findings of the study presented here are another step towards understanding the interplay between insulin-mediated signaling and diseases caused by WNV and other flaviviruses. More broadly, further studies on the interplay of multiple arms of immunity in infectious disease can reveal how the components synergize and compensate for one another to combat infection while reducing the risk of pathogenic escape variants to form.

## Materials and methods

### Cell culture and virus

Baby hamster kidney-21 (BHK-21) (ATCC, CCL-10) and HepG2 (ATCC, HB-8065) cells were cultured in DMEM supplemented with 10% fetal bovine serum (FBS) and maintained at 37°C in 5% CO_2_. *Culex quinquefasciatus* Hsu cells were cultured in L-15 media supplemented with 10% FBS and 10% triphosphate buffer. These cells were maintained at 28°C in 5% CO_2_. West Nile virus-Kunjin strain MRM16 (WNV-Kun) was gifted by R. Tesh and propagated in Vero cells. West Nile virus strain 385-99 (WNV-NY99) was obtained from BEI Resources, NIAID, NIH (NR-158) and propagated in Vero cells. All experiments with a specific virus type utilized the same stock.

### Fruit fly infections

Knockdown of *InR* was achieved by crossing *y^1^v^1^;P{TRiP.JF01482}attP2* (InR dsRNA cassette, Bloomington *Drosophila* Stock Center (BDSC) 31037) with y^1^w*;P{Act5C-GAL4}25FO1/CyO,y^+^ (Actin driver, BDSC 4144). Sibling offspring not expressing the actin driver were used as controls. *Ilp7^1^* mutant flies (*w^1118^;TI{w^+mW.hs^=TI}Ilp7^1^*, BDSC 30887) were compared to *w^1118^* controls (BDSC 5905). Oregon-R-C flies (BDSC 5) were also used. Flies are negative for *Wolbachia* infection. Flies were kept on standard cornmeal food (Genesee Scientific #66–112) at a temperature of 25°C with 65% relative humidity and a 12-hour light/dark cycle. The adult flies used for experiments were female and aged between 2 to 7 days post-eclosion. Bovine insulin (Sigma 10516) was incorporated into the food to achieve a final concentration of 10 μM. Flies were intrathoracically injected with 2,000 PFU of WNV-NY99 or PBS vehicle control, as previously described (Ahlers et al., 2019).

### Virus titration

Virus replication in cells and flies was measured using a standard plaque assay on BHK-21 cells as previously described (Mead et al., 2024). Hsu cell culture supernatant from three biological replicates per condition was collected at 5 days post-infection and stored at -80°C. At 5 and 10 days post-infection, three sets of five flies were collected from each treatment condition. Flies were homogenized in 50 μl Phosphate-Buffered Saline (PBS) and stored at -80°C. Cell culture supernatant or fly homogenate was serially diluted in DMEM with 2% FBS and plated on a 12-well plate of BHK21 cells at 1.5 × 10^5^ cells/well. Plates were incubated for 2 h at 37°C/5% CO_2_ and rocked every 15 min. Wells were overlayed with 4% low melting point agarose (Invitrogen 16520050) for a final concentration of 0.75% agarose and 4% FBS in DMEM. Plates were incubated for four days at 37°C/5% CO_2_ before visualization with 0.1% crystal violet (Fisher 548-62-9).

### Serial passage

Hsu cells were seeded onto a 6-well plate at a density of 10^6^ cells per well and incubated overnight. At 24 hours post-seeding, the cell media in each well was aspirated and replaced with either 0 μM or 1.7 μM insulin media, depending on the treatment group. After 24 hours of incubation in the chosen media, the cells were infected with WNV at MOI 0.001 PFU/cell. The plates were incubated with virus for 2 hours before aspirating the infection media and replaced with 2% FBS L-15 media. Infected Hsu plates were incubated for 5 days before collection of cell media. Cells adhered to the plate were lysed in 300 μL of Trizol and stored at -80°C to preserve RNA.

### HepG2 infection

HepG2 cells were seeded onto a 24-well plate at a density of 7.5 x 10^5^ cells per well and incubated for 48 hours. After the incubation period, the cells were infected with WNV at MOI 0.001 PFU/cell. The plates were incubated with virus for 1 hour before aspirating the infection media and replaced with 2% FBS DMEM media.

### Immunoblotting

Protein lysate was collected by applying 100 μL of RIPA buffer (Ahlers et al., 2019) to each well of infected HEPG2 cells or by homogenizing five mosquitoes in 100 μL RIPA buffer. The concentration of protein lysate was assessed by BCA assay. 5 μg of protein was diluted in 2x Laemmli and heated to 95°C for 5 minutes. The prepared samples were analyzed by SDS-PAGE using 10% acrylamide gel followed by transfer to a PVDF membrane. Blots used to target Akt (1:2,000) (Cell Signaling 4691) were blocked in 5% non-fat dairy milk (NFDM) in Tris-buffered saline and 0.1% Tween (TBST) before application of primary antibodies. Blots used to target P-Akt (1:1,000; Cell Signaling 4060) or WNV envelope (1:250, Novus Biologicals NBP2-52709) were blocked in 5% BSA in TBST before application of primary antibodies. Blots were incubated with primary antibodies overnight at 4°C followed by 2-hour incubation with HRP-conjugated secondary anti-rabbit or -mouse IgG-HRP conjugate antibodies (1:10,000; Promega W401B, W402B) at room temperature. For actin blotting (1:10,000; Sigma A2066), PVDF membranes were stripped with gentle rocking for 30 minutes. The procedure for actin blotting was similar to Akt blots. Densitometry was performed using ImageJ.

### Workflow for sanger sequencing

RNA was isolated (ZymoResearch R2050) from three biological replicates of infected Hsu cells at passages 5 and 9 (**Figure 2**) and 1 μg of RNA was used to prepare cDNA (BioRad 170-8891). The cDNA was used as the template for various PCR reactions targeting the 4 genes of interest - E, NS2B, NS3 and NS5 (**Table 2**).

**Table 2:** Primer list.

Target gene	Primer title	Primer sequence
ENV	WNV_4_LEFT	GCACCAAGGCCACAAGGTATTT
	WNV_9_RIGHT	TCCATCCAAGCCTCCACATCAT
NS2B	WNV_16_LEFT	ATCAGGGAGAAGAGGAGTGCAG
	WNV_17_RIGHT	GCCCACGAGTCATGATCCTGTA
NS3	WNV_18_LEFT	TGCCCTCAGTAGTTGGATTTTGG
	WNV_20_RIGHT	GTCGGTGAAATGAGCCTCATCC
	WNV_21_LEFT	CGCAGTGCCCAGAGAACATAAT
	WNV_24_RIGHT	CCCAGAACCTCAATGAGCCCTA
NS5	WNV_29_LEFT	TGCAGAAGAAAGTTGGACAGATCA
	WNV_33_RIGHT	CGTTGAGCACGTACTTCACTCC
	WNV_34_LEFT	CGAATGTTACCACCATGGCCAT
	WNV_39_RIGHT	TCTACAGTACTGTGTCCTCAACCA

The genes coding for NS3 and NS5 were amplified in two parts by overlapping primer pairs. The primers targeting these genes were selected from a phylogenetic study of WNV-NY99 in Arizona which used an array of primers to sequence the whole virus genome (Hepp et al., 2018). The products from these PCR reactions were purified (NEB T1030S). The purified products were sent to Eurofins for Sanger sequencing. Forward and reverse sequencing reactions were prepared for the genes of each biological replicate. The forward and reverse sequence files were used to assemble complete gene sequences using the ‘Assemble contigs’ function in SnapGene (v7.1.1). These complete sequences were aligned to the genome of the virus used for the first passage and analyzed for mutations.

### Mosquito rearing

Female *Cx. quinquefasciatus* (BEI Resources NR-43025) were reared as described previously (Kauffman et al., 2017), with modifications. Briefly, mosquitoes were maintained under a 12 h light-dark cycle and at 28°C and 80% humidity. Adult mosquitoes were housed in a 1-gallon polypropylene Nalgene container with mesh screening and maintained on 10% sucrose-soaked cotton balls. For egg production, the adult females (~7 days old) were allowed to feed on defibrinated chicken blood overnight using an artificial mosquito feeder (Chemglass Life Sciences). Mosquitoes were provided with oviposition substrates, clear cups containing distilled water with soaked filter paper, 3 days following the blood meal. The collected egg rafts were washed into larval flats with distilled water and allowed to hatch in the same pans, with daily supplementation with fish food until pupation. Experimental mosquitoes (7 to 15 days old) were transferred to a 2.5 L polypropylene Nalgene container with mesh screening at least 24 h prior to the beginning of an experiment.

### Mosquito infections

WNV-NY99 or -Kun was mixed with defibrinated chicken blood, resulting in a final concentration of 10^7^ PFU/mL. For WNV-Kun infections, the bloodmeal was supplemented with HEPES buffer or 170 pM insulin, as described previously (Ahlers et al., 2019). Female *Cx. quinquefasciatus*, bred for three generations, deprived of sucrose for 12 h, and then fed using an artificial mosquito feeder (Chemglass Life Sciences). Females were kept in 2.5 L cartons with constant access to 10% sucrose, and collected at 3 or 10 dpi for analysis. Females that did not feed were excluded from further analysis.

### RNA extraction and RNAseq

Mosquito tissues were lysed with gentle disruption in Trizol Reagent (ThermoFisher 15596), RNA was isolated by column purification (ZymoResearch R2050) and DNA was removed (ThermoFisher 18068). Following RNA extraction, library preparation and RNAseq was performed as previously described (Trammell et al., 2023). RNA sequencing was performed at the Spokane Genomics CORE at Washington State University-Spokane in Spokane, WA, USA.

### Gene set enrichment analysis

Gene Set Enrichment Analysis (GSEA) was performed using RNAseq datasets and their associated p values as previously described (Subramanian et al., 2005). The GSEA used transcript levels for each experimental condition normalized to 0 µM insulin from the RNAseq analysis. GO categories were selected based on GSEA p value (p < 0.05).

### Molecular modeling of WNV NS3

The NS3 of WNV is made up of two domains: a helicase and a protease. The crystal structure of WNV-Kun helicase is available (PDB id-2QEQ) without bound form of the ADP nucleotide. To generate the full NS3 protein, a homology model of WNV-Kun helicase-protease was generated using the structure of NS3 protease-helicase from dengue virus (PDB ID-2WHX) using the Prime-structure prediction wizard of Schrödinger (2020-4). The conformation of nucleotide ADP was transferred to the homology model from NS3 protease-helicase from dengue virus and loops of the homology model were refined using Prime Module of Schrödinger (2020-4).

Desmond, a molecular dynamics (MD) engine of Schrödinger (2020-4), was used to execute the MD calculations for predicting the stability of WNV-Kun helicase-protease-ADP complex. Two separate MD simulations were performed: 1) WNV-Kun helicase-protease-ADP complex, 2) Mutant K84I WNV-Kun helicase-protease-ADP complex. The complexes were embedded in the TIP3P water model of orthorhombic box and were neutralized with the counter ions and physiological salt concentration kept at 0.15 M of NaCl. The system was specified on periodic boundary conditions, the particle mesh Ewald (PME) method for electrostatics, a 10 Å cutoff for Lennard–Jones interactions and SHAKE algorithm for limiting movement of all covalent bonds involving hydrogen atoms. OPLS3e forcefield was used to prepare the systems.

The energy was minimized using hybrid method of steepest descent (10 steps) and the LBFGS (limited-memory Broyden–Fletcher–Goldfarb–Shanno) algorithm with a maximum of 2000 steps with solute restrained, followed by similar energy minimization for 2000 steps without solute restraints. Then 12 ps simulation was carried out in NVT (Constant volume and temperature) ensemble for restraining nonhydrogen solute atoms at 10 K temperature was repeated and then followed by 12 ps simulation in the NPT (constant pressure and temperature) ensemble of temperature 10 K for restraining nonhydrogen solute atoms. About 24 ps simulation in the NPT ensemble were restrained with solute nonhydrogen atoms at temperature 300 K and 24 ps simulation in the NPT ensemble at temperature 300 K with no restraints. Berendsen thermostats and barostats were used to control the temperatures and pressures during the initial simulations. The relaxed system was subjected to simulation time of 50 ns with a time step of 2 fs in the NPT ensemble using a Nose–Hoover thermostat at 300 K and Martyna–Tobias–Klein barostats at 1.01 bar pressure. Every trajectory was recorded with a time interval of 100 ps.

MM-GBSA was calculated for MD trajectories of both complexes 1) WNV-Kun helicase-protease-ADP complex, 2) Mutant K84I WNV-Kun helicase-protease-ADP complex using the Prime module of Schrödinger (2020-4) to calculate binding affinity (ΔΔG_bind,ADP_) of ADP. The difference between the former and latter provided the binding affinity change (ΔΔG_bind,ADP_) of ADP due to the K84I mutation. Residue scanning of Schrödinger (2020-4) was performed on snapshots of MD trajectory of WNV-Kun helicase-protease-ADP complex, to check the stability (ΔΔG_fold,ADP_) of the structure due to mutation K84I.

### Quantification and statistical analyses

Results shown are representative of at least three independent experiments. Data points in dot plots and bar graphs represent a biological replicate of an individual well of cells (**Figures 1C–F**, **2**), a pool of five flies (**Figure 1G**), or an immunoblot (**Figures 3**, **4**). Statistical analyses were completed using GraphPad Prism. Two-tailed unpaired t-tests assuming unequal variance were utilized to compare normally distributed pairwise quantitative data. Two-way ANOVA with Šidák correction was used to compare multivariate data. All error bars represent standard error of the mean. Survival curves (**Figures 1A, B**) represent three replicate experiments per condition pooled together and analyzed by the log-rank (Mantel-Cox) test using GraphPad Prism to determine P values between infected genotypes.

## Data Availability

The datasets presented in this study can be found in online repositories. The names of the repository/repositories and accession number(s) can be found below: https://www.ncbi.nlm.nih.gov/geo/query/acc.cgi?acc=GSE269077.
