# Supplementary material for: Sustained antiviral insulin signaling during West Nile virus infection results in viral mutations
Source: Front Cell Infect Microbiol. 2024 Nov 27;14:1492403. doi: 10.3389/fcimb.2024.1492403 (PMC11631865; doi:10.3389/fcimb.2024.1492403)
Supplement: Supplementary file 1 [file Table1.docx]

**Supplementary Table 1 – Gene IDs and Log_2_ fold change data (normalized to mock infection) of heatmap presented in Figure 5B.**

| **VectorBase Gene ID** | **RefSeq Gene ID** | **170 pM insulin** | **0 pM insulin** |
| --- | --- | --- | --- |
| CPIJ010028 | XM_001851335.1 | 2.030940475 | -0.899318876 |
| CPIJ007014 | XM_001848385.1 | 2.02240012 | -0.441494954 |
| CPIJ001441 | XM_001842948.1 | 1.584962501 | -10 |
| CPIJ015860 | XM_001865926.1 | 1.187085553 | 1.03542949 |
| CPIJ017980 | XM_001868047.1 | 1.117530876 | -1.276805975 |
| CPIJ005552 | XM_001847741.1 | 1.103622631 | 0.372880186 |
| CPIJ002102 | XM_001843318.1 | 1.087110664 | 0.086054647 |
| CPIJ015415 | XM_001865981.1 | 1.036696532 | 0.778605324 |
| CPIJ005583 | XM_001847771.1 | 1.03055292 | -0.085779863 |
| CPIJ010035 | XM_001851342.1 | 0.970133305 | -1.927164135 |
| CPIJ007940 | XM_001849690.1 | 0.961797546 | -1.009847786 |
| CPIJ018609 | XM_001868748.1 | 0.937264245 | -0.302092913 |
| CPIJ017329 | XM_001867443.1 | 0.936878188 | 0.177125356 |
| CPIJ007378 | XM_001848774.1 | 0.908322886 | 0.156420153 |
| CPIJ005864 | XM_001847637.1 | 0.895582517 | -0.275253172 |
| CPIJ002909 | XM_001844475.1 | 0.820972791 | -0.467235578 |
| CPIJ002452 | XM_001844009.1 | 0.795787595 | 0.681594972 |
| CPIJ008340 | XM_001849552.1 | 0.739319094 | 0.10437423 |
| CPIJ007693 | XM_001849535.1 | 0.678071905 | -0.962436587 |
| CPIJ002728 | XM_001844259.1 | 0.670885836 | -0.506878781 |
| CPIJ007483 | XM_001848601.1 | 0.606287546 | -0.113833923 |
| CPIJ006168 | XM_001847560.1 | 0.598809014 | -0.349813904 |
| CPIJ003743 | XM_001845264.1 | 0.597141887 | -0.101061528 |
| CPIJ005611 | XM_001847011.1 | 0.564587951 | -0.245281524 |
| CPIJ012886 | XM_001863192.1 | 0.561098561 | -0.0420128 |
| CPIJ019106 | XM_001869362.1 | 0.554156454 | 0.612976877 |
| CPIJ013692 | XM_001863990.1 | 0.480625841 | 1.070389328 |
| CPIJ014786 | XM_001865073.1 | 0.459072029 | -0.326271385 |
| CPIJ013698 | XM_001863996.1 | 0.440972056 | -0.473311395 |
| CPIJ011068 | XM_001861429.1 | 0.41824435 | 0.326230146 |
| CPIJ019706 | XM_001870034.1 | 0.405614245 | 0.422682823 |
| CPIJ011277 | XM_001861524.1 | 0.401957672 | 0.077519631 |
| CPIJ010110 | XM_001854594.1 | 0.301169535 | 0.231541858 |
| CPIJ009982 | XM_001853180.1 | 0.282752929 | 0.276423783 |
| CPIJ013852 | XM_001864524.1 | 0.256966485 | -0.331810161 |
| CPIJ008237 | XM_001849913.1 | 0.246559269 | 0.278934877 |
| CPIJ009957 | XM_001851386.1 | 0.238063823 | -0.533486283 |
| CPIJ018234 | XM_001868371.1 | 0.234465254 | -0.490512924 |
| CPIJ010349 | XM_001870954.1 | 0.233306478 | -0.033689224 |
| CPIJ008203 | XM_001849401.1 | 0.213830233 | -0.449913595 |
| CPIJ015133 | XM_001865284.1 | 0.202058928 | -0.428230833 |
| CPIJ016072 | XM_001867089.1 | 0.187278568 | -0.806089955 |
| CPIJ007967 | XM_001849869.1 | 0.179578552 | 0.022761338 |
| CPIJ013834 | XM_001863981.1 | 0.177598741 | 0.116618062 |
| CPIJ004780 | XM_001846338.1 | 0.148331909 | 0.477250322 |
| CPIJ013823 | XM_001863970.1 | 0.123293463 | -0.291329615 |
| CPIJ002908 | XM_001844474.1 | 0.086951017 | -0.244034256 |
| CPIJ005732 | XM_001847103.1 | 0.079389326 | -0.509727774 |
| CPIJ013850 | XM_001864522.1 | 0.064261998 | 0.167241497 |
| CPIJ016121 | XM_001866386.1 | 0.038474148 | -0.829083796 |
| CPIJ010574 | XM_001870775.1 | 0.020291404 | -1.245112498 |
| CPIJ007327 | XM_001848643.1 | 0.018434892 | -0.310340121 |
| CPIJ005149 | XM_001846877.1 | 0.015767316 | 1.14543044 |
| CPIJ003330 | XM_001845004.1 | -0.010454358 | -0.108438552 |
| CPIJ003821 | XM_001845342.1 | -0.052196211 | 0.32305476 |
| CPIJ012681 | XM_001862909.1 | -0.055589861 | -0.512410211 |
| CPIJ015287 | XM_001865497.1 | -0.068587085 | 0.772085294 |
| CPIJ018984 | XM_001869221.1 | -0.140984732 | -0.70148715 |
| CPIJ010278 | XM_001870646.1 | -0.141868716 | -0.373161294 |
| CPIJ014935 | XM_001864984.1 | -0.161075459 | -0.193212145 |
| CPIJ016188 | XM_001866241.1 | -0.166706854 | -0.467154596 |
| CPIJ012803 | XM_001862854.1 | -0.167515143 | 0.100404942 |
| CPIJ019411 | XM_001869676.1 | -0.167599948 | -1.259386629 |
| CPIJ004082 | XM_001845660.1 | -0.169680994 | 0.096785063 |
| CPIJ017247 | XM_001867601.1 | -0.193681125 | -1.025237291 |
| CPIJ001216 | XM_001842833.1 | -0.22474145 | 0.567527907 |
| CPIJ007319 | XM_001848635.1 | -0.231401121 | -0.475226459 |
| CPIJ002099 | XM_001843315.1 | -0.251323423 | -0.052974465 |
| CPIJ012682 | XM_001862910.1 | -0.264423616 | 1.235378063 |
| CPIJ009979 | XM_001853168.1 | -0.305668681 | -1.093109404 |
| CPIJ005296 | XM_001846622.1 | -0.317917688 | -0.764769999 |
| CPIJ009164 | XM_001850600.1 | -0.327050328 | 0.069425282 |
| CPIJ013497 | XM_001863697.1 | -0.335169379 | -0.013778086 |
| CPIJ011908 | XM_001862086.1 | -0.337358135 | -1.268051138 |
| CPIJ005774 | XM_001847206.1 | -0.395125644 | -0.509295089 |
| CPIJ007526 | XM_001849288.1 | -0.419781083 | -0.811701246 |
| CPIJ008049 | XM_001850070.1 | -0.430355445 | 1.354554196 |
| CPIJ004831 | XM_001846389.1 | -0.45106317 | -0.536795218 |
| CPIJ012257 | XM_001862570.1 | -0.458069515 | 0.205510705 |
| CPIJ016926 | XM_001867047.1 | -0.470388732 | -0.079302891 |
| CPIJ011585 | XM_001862177.1 | -0.538908758 | -0.60820205 |
| CPIJ006141 | XM_001847533.1 | -0.550920927 | 1.017465142 |
| CPIJ002124 | XM_001843340.1 | -0.595565032 | -0.572996266 |
| CPIJ017452 | XM_001867575.1 | -0.603717066 | -0.299404603 |
| CPIJ003763 | XM_001845284.1 | -0.62572537 | -0.695798074 |
| CPIJ014604 | XM_001864814.1 | -0.728075993 | -0.740576026 |
| CPIJ003423 | XM_001845141.1 | -0.744173351 | -1.261120708 |
| CPIJ000514 | XM_001842132.1 | -0.768318013 | -0.593923368 |
| CPIJ005508 | XM_001846933.1 | -0.845628921 | -0.353764182 |
| CPIJ014605 | XM_001864815.1 | -0.941083182 | -0.597975623 |
| CPIJ018903 | XM_001869081.1 | -0.944504887 | -0.055742552 |
| CPIJ005939 | XM_001847335.1 | -0.944876005 | -0.484326233 |
| CPIJ005519 | XM_001846944.1 | -1 | 1 |
| CPIJ010815 | XM_001858558.1 | -1.053182281 | -1.122763824 |
| CPIJ007660 | XM_001849136.1 | -1.066251082 | 0.25415205 |
| CPIJ005703 | XM_001847074.1 | -1.066925473 | -0.159382624 |
| CPIJ018795 | XM_001868928.1 | -1.094779283 | 0.524126459 |
| CPIJ012024 | XM_001862256.1 | -1.104555931 | 0.067355268 |
| CPIJ001668 | XM_001843156.1 | -1.181267688 | 0.694249259 |
| CPIJ015113 | XM_001865114.1 | -1.225627495 | -0.501093379 |
| CPIJ013244 | XM_001863906.1 | -1.413152855 | 0.355828502 |
| CPIJ005487 | XM_001846912.1 | -1.450893652 | -0.063886879 |
| CPIJ014664 | XM_001865766.1 | -1.454747209 | -0.722992652 |
| CPIJ000699 | XM_001842212.1 | -1.500720114 | 0.204959897 |
| CPIJ011725 | XM_001862047.1 | -1.521134377 | -0.327027921 |
| CPIJ001508 | XM_001843757.1 | -1.608809243 | 1.314510623 |
| CPIJ004179 | XM_001845851.1 | -1.699295176 | -2.377271129 |
| CPIJ004171 | XM_001845843.1 | -1.790772038 | -0.16432043 |
| CPIJ012957 | XM_001863080.1 | -1.793070696 | 0.903275229 |
| CPIJ011335 | XM_001862083.1 | -2 | -10 |
| CPIJ011899 | XM_001862063.1 | -2 | 0.847996907 |
| CPIJ005352 | XM_001846742.1 | -2.047753131 | -1.008988783 |
| CPIJ018118 | XM_001868250.1 | -2.056810921 | -0.115873791 |
| CPIJ004954 | XM_001846423.1 | -2.085575732 | 2.830718915 |
| CPIJ011963 | XM_001862027.1 | -2.559885149 | 0.531593851 |
| CPIJ017505 | XM_001867851.1 | -2.662965013 | -10 |
